# Identification of enzymes involved in oxidation of phenylbutyrate

**DOI:** 10.1194/jlr.M075317

**Published:** 2017-04-28

**Authors:** Neža Palir, Jos P. N. Ruiter, Ronald J. A. Wanders, Riekelt H. Houtkooper

**Affiliations:** Laboratory Genetic Metabolic Diseases, Academic Medical Center, University of Amsterdam, 1105 AZ Amsterdam, The Netherlands

**Keywords:** β-oxidation, cells and tissues, dehydrogenases, enzymology, fatty acid/oxidation, lipid biochemistry, phenylacetate, cell metabolism, coenzyme A

## Abstract

In recent years the short-chain fatty acid, 4-phenylbutyrate (PB), has emerged as a promising drug for various clinical conditions. In fact, PB has been Food and Drug Administration-approved for urea cycle disorders since 1996. PB is more potent and less toxic than its metabolite, phenylacetate (PA), and is not just a pro-drug for PA, as was initially assumed. The metabolic pathway of PB, however, has remained unclear. Therefore, we set out to identify the enzymes involved in the β-oxidation of PB. We used cells deficient in specific steps of fatty acid β-oxidation and ultra-HPLC to measure which enzymes were able to convert PB or its downstream products. We show that the first step in PB oxidation is catalyzed solely by the enzyme, medium-chain acyl-CoA dehydrogenase. The second (hydration) step can be catalyzed by all three mitochondrial enoyl-CoA hydratase enzymes, i.e., short-chain enoyl-CoA hydratase, long-chain enoyl-CoA hydratase, and 3-methylglutaconyl-CoA hydratase. Enzymes involved in the third step include both short- and long-chain 3-hydroxyacyl-CoA dehydrogenase. The oxidation of PB is completed by only one enzyme, i.e., long-chain 3-ketoacyl-CoA thiolase. Taken together, the enzymatic characteristics of the PB degradative pathway may lead to better dose finding and limiting the toxicity of this drug.

Sodium 4-phenylbutyrate (PB) is a phenolic short-chain fatty acid that is prescribed as a treatment for patients with urea cycle disorders. These patients suffer from hyperammonemia resulting from genetic defects in the various steps of the urea cycle (1, 2). An alternative pathway to remove excess nitrogen involves conjugation of glutamine with the PB-derived metabolite, phenylacetate (PA), leading to the formation of phenylacetylglutamine (3). Even though PA was initially prescribed as a treatment, the terrible odor and higher toxicity of PA (4) led to a preferential treatment with its precursor PB, which is activated to its CoA-ester and then metabolized to phenylacetyl-CoA (PA-CoA) by fatty acid β-oxidation (5).

In addition to urea cycle disorders, PB shows promise as a treatment for patients with rare pyruvate dehydrogenase complex (PDHC) deficiency (6) or branched-chain keto acid dehydrogenase complex (BCKDC) deficiency, the latter causing so-called maple syrup urine disease (7). Patients with maple syrup urine disease accumulate branched-chain amino acids and the corresponding branched-chain α-keto acids; but upon treatment with PB, they show reduced concentrations of both metabolites in blood (7). PB prevents BCKDC from inactivation by BCKDC kinase, thereby enhancing overall BCKDC activity (7). A similar effect of PB was identified in patients suffering from PDHC deficiency (6). PB enhances the activity of PDHC by directly inhibiting pyruvate dehydrogenase kinase, the enzyme that inactivates PDHC through phosphorylation (6). Finally, even though the modes of action are not completely elucidated, PB also exerts positive effects on other clinical conditions. It decreases very-long-chain fatty acids in cultured cells from X-linked adrenoleukodystrophy patients by increasing β-oxidation of very-long-chain fatty acids in the peroxisome (8). PB has also shown beneficial effects for the treatment of cystic fibrosis (9), Alzheimer’s disease (10), Huntington’s disease (11), type 2 diabetes (12), and different types of cancer (13–17).

The efficacy and toxicity of PB or its downstream products is dependent on its metabolism. The main site for oxidation of fatty acids with <20 carbon atoms is the mitochondrion. Acyl-CoAs undergo β-oxidation consisting of four sequential enzymatic steps: dehydrogenation, hydration, a second dehydrogenation, and thiolytic cleavage, thereby shortening the acyl-CoA by two carbons and producing acetyl-CoA, which can enter the citric acid cycle (18).

The role of fatty acid β-oxidation in the breakdown of PB was already described over a century ago, when it was found that feeding ω-phenylbutyric acid and ω-phenylvaleric acid to dogs led to the excretion of phenylacetic acid and hippuric acid in urine (19). Despite this early discovery, the metabolic degradation pathway of PB has remained largely unknown. Indeed, the first step in PB β-oxidation was resolved only recently, demonstrating that purified medium-chain acyl-CoA dehydrogenase (MCAD) can catalyze the dehydrogenation of 4-phenylbutyryl-CoA (PB-CoA) (20). In line with this, cell extracts from MCAD-deficient patients displayed no detectable activity with PB-CoA as a substrate (20).

Here, we confirmed that MCAD is the only enzyme responsible for the first step of β-oxidation of PB-CoA. In addition, we used fibroblasts deficient in other steps of β-oxidation to identify the enzymes capable of completing the remaining three steps in PB oxidation. We show that short-chain enoyl-CoA hydratase (SCEH; also known as crotonase), long-chain enoyl-CoA hydratase (LCEH), and 3-methylglutaconyl-CoA hydratase (3-MGH) can catalyze the second step. The third step is catalyzed by both short-chain 3-hydroxyacyl-CoA dehydrogenase (SCHAD) and long-chain 3-hydroxyacyl-CoA dehydrogenase (LCHAD). The oxidation cycle is completed by long-chain 3-ketoacyl-CoA thiolase (LCKAT). Altogether, our data demonstrate the importance of specific fatty acid β-oxidation enzymes in regulating PB as a therapeutic agent.

## MATERIALS AND METHODS

### PB-CoA and PA-CoA synthesis

PB-CoA was synthesized as described previously (21). Briefly, 36 μmol of phenylbutyric acid were dissolved in 1.4 ml dichloromethane:tetrahydrofuran (5:2) and 40 μl triethylamine (1 M in dichloromethane) and left to incubate for 10 min with constant stirring. Thereafter, 40 μl of ethyl chloroformate (1 M in dichloromethane) were added and incubated for 45 min at room temperature under nitrogen and continuous mixing. The product was dried completely with nitrogen and the dry residue was dissolved in 0.5 ml t-butanol. To the mixture, 0.5 ml of CoA (80 mM dissolved in 0.4 M potassium hydrogen carbonate) were added and allowed to react at room temperature for 30 min under continuous mixing. The reaction was stopped by addition of 100 μl of hydrochloric acid (0.1 M) and the reaction mixture was dried under a stream of nitrogen. The dry residue was resuspended in MES (20 mM, pH 6). PB-CoA was purified by HPLC using a SPLC-18-DB 5 μm column (250 × 10 mm) and a linear gradient (10–15%) of buffer B into buffer A at a flow rate of 3 ml/min over 30 min. For buffer A, sodium phosphate buffer (16.9 mM, pH 6.9) was mixed with acetonitrile in a 9:1 ratio. Buffer B had the same components, but in a ratio of 3:7. The PB-CoA fraction was collected, dried, and dissolved in MES (20 mM, pH 6). To form PA-CoA, the same protocol was followed as for PB-CoA synthesis, only replacing phenylbutyric acid with phenylacetic acid.

### Synthesis of 2-hexadecenoyl-CoA

The 2-hexadecenoyl-CoA was synthesized from hexadecanoyl-CoA (10 μmol) using 0.5 U acyl-CoA oxidase (Wako; 019-10841) in a medium (100 ml final volume) containing 100 mM TRIS buffer (pH 8.0). The reaction was followed by measuring the increase of the extinction at 263 nm on a spectrophotometer. The reaction was stopped when it reached a plateau by addition of hydrochloric acid (0.5 M final concentration). The product was allowed to precipitate for 30 min on ice and collected by centrifugation (10 min, 4,000 *g*). The pellet was dissolved in 20 mM MES buffer at pH 6.

### Synthesis of standards

#### Synthesis of 4-phenyl-2-butenoyl-CoA.

The 4-phenyl-2-butenoyl-CoA (PB:1-CoA) was synthesized from PB-CoA using acyl-CoA oxidase (Wako; 019-10841) in a medium containing 100 mM TRIS buffer (pH 8.0). The reaction was followed by measuring the increase of the extinction at 263 nm on a spectrophotometer. The reaction was stopped when it reached a plateau by addition of hydrochloric acid (0.5 M final concentration) and pH was adjusted to pH 6 with 2 M KOH/0.6 M MES.

#### Synthesis of 4-phenyl-3-hydroxybutyryl-CoA.

To form 4-phenyl-3-hydroxybutyryl-CoA (PHB-CoA), PB-CoA was incubated with acyl-CoA oxidase and SCEH (expressed in *Escherichia coli* from pET19B-6×-HIS-SCEH and purified from a Ni-Sepharose column) in 100 mM TRIS buffer (pH 8.0). The reaction was stopped as described above.

#### Synthesis of 4-phenyl-3-ketobutyryl-CoA.

To synthesize 4-phenyl-3-ketobutyryl-CoA (PKB-CoA), PB-CoA was incubated with acyl-CoA oxidase, SCEH, and 3-hydroxyacyl-CoA dehydrogenase (Sigma; H-7384) in a medium containing 100 mM TRIS buffer (pH 8.0), 1 mM NAD, 5 mM pyruvate, and lactate dehydrogenase.

### In vitro PB-CoA oxidation

Fibroblast pellets were homogenized by sonication (three cycles of 15 s at 7–8 W with 60 s time intervals under continuous cooling on ice water) in PBS containing 50 μM FAD. The incubation mixture (total volume of 50 μl) consisted of 100 mM TRIS (pH 8), 260 μM PB-CoA, 1 mM ferrocenium hexafluorophosphate, 50 μM FAD, 1 mM NAD, 5 mM pyruvate, 10 mM oxaloacetate, 50 μM CoA, 0.75 U lactate dehydrogenase, and 7.5 U citrate synthase. The reaction was started by addition of 25 μg of fibroblast protein and allowed to proceed of 30 min at 37°C. Reactions were stopped with 5 μl 2 M HCl and the mixture was kept on ice for 5 min and adjusted to pH 6 with 5 μl 2 M KOH/0.6 M MES. Before centrifugation (11,000 *g*, 5 min, 4°C), methanol was added to the samples and the acyl-CoA esters were immediately analyzed by ultra-(U)HPLC.

### Quantification of acyl-CoA species by UHPLC

The mobile phases used were buffer A and buffer B. For buffer A, 50 mM of potassium phosphate buffer (pH 5.3) were mixed with methanol in a ratio of 9:1. Buffer B had the same components in a ratio of 1:1. All the samples were analyzed on an Ultimate 3000 UHPLC system from Thermo Scientific with a Waters Acquity HSS C18 1.8 μm column (2.1 × 100 mm) at a wavelength of 260 nm and a linear gradient of 50–100% of buffer B in 19 min.

### Antibodies

SCEH and SCHAD polyclonal antibodies were raised in rabbits by injecting crotonase and β-hydroxyacyl-CoA dehydrogenase from bovine liver (Sigma), respectively. Pre-immune serum from the same rabbits was collected prior to injection of the proteins.

### Immunoprecipitation

Protein A Sepharose was added to 5 ml of PBS and left to swell for an hour. Beads were centrifuged (4,000 *g*, 4°C, 1 min) and the supernatant was removed. Beads were washed three times with 5 ml PBS + Triton X-100 (1 g/l) + BSA (10 g/l) and five times with 5 ml PBS + Triton X-100 (1 g/l) (PBS-T). SCEH, SCHAD, and pre-immune rabbit serum were serially diluted with PBS-T in a 1:3 ratio. To 50 μl (bed volume) protein A Sepharose, 100 μl of diluted serum were added and incubated overnight at 4°C. The next day mitochondrial trifunctional protein (MTP)-deficient fibroblasts were homogenized in PBS-T and sonicated (three cycles of 15 s at 7–8 W with 60 s time intervals under continuous cooling on ice water). Lysates were cleared by centrifugation (5,000 *g*, 10 min, 4°C). Protein A Sepharose beads with serum were washed five times with 1 ml PBS-T and 200 μl of cell lysate were added and left to incubate at 4°C for 2 h. After incubation, the mixtures were centrifuged (4,000 *g*, 1 min, 4°C) and the supernatants were used to quantify the enzyme activity. To remove two enzymes from the same lysate, Protein A Sepharose beads coated with SCEH and 3-MGH antibodies were prepared separately and then combined just before adding the cell lysate. Effective removal (>92%) was confirmed using specific enzyme activity measurements.

### Enzyme activity measurements

#### LCEH and SCEH activity measurement.

LCEH and SCEH activity were measured in a mixture (50 μl final volume) containing 100 mM TRIS (pH 8.0) using 0.5 mM 2-hexadecenoyl-CoA or 2-butenoyl-CoA, respectively, as substrate. The reaction was started by addition of lysate (10 μg/ml final concentration) and was subsequently incubated at 37°C for 5 min. The reaction was stopped with 5 μl 2 M HCl and the mixture was neutralized with 5 μl 2 M KOH/0.6 M MES. Proteins were precipitated (11,000 *g*, 4°C, 5 min) and the cleared supernatant was analyzed by UHPLC to quantify 3-hydroxyhexadecanoyl-CoA (for LCEH) or 3-hydroxybutyryl-CoA (for SCEH).

#### The 3-MGH activity measurement.

The 3-MGH activity was measured in a mixture (100 μl final volume) containing 100 mM TRIS (pH 7.4), 2.4 mM 3-hydroxy-3-methylglutaryl-CoA, 10 mM EDTA, and 1 mg/ml BSA. The reaction was started by addition of lysate (75 μg/ml final concentration). After 30 min incubation at 37°C, the reaction was stopped with 10 μl 2 M HCl, neutralized with 2 M KOH/0.6 M MES, and centrifuged (11,000 *g*, 4°C, 5 min). The cleared supernatant was analyzed by HPLC to quantify 3-methylglutaconyl-CoA.

#### SCHAD activity measurement.

SCHAD activity was measured spectrophotometrically as described before (21).

#### Gel filtration.

Gel filtration was performed as described previously (22).

## RESULTS

To identify the enzymes responsible for the degradation of PB-CoA, we used human fibroblasts that were deficient in one of the steps of β-oxidation (**Table 1**). Cell extracts were incubated with PB-CoA and the formation of downstream β-oxidation products was analyzed using UHPLC. If the enzyme responsible for the conversion of PB-CoA or one of its intermediates was deficient, the product of that step, as well as its downstream products, should not be formed.

**TABLE 1.** Enzyme activities in human fibroblast homogenates

Defective Enzyme/Gene Symbol	Cell Line Code	Genotype of Defective Enzyme*^a^*	Activity of Defective Enzyme (nmol/min/mg)	Activity in Controls (nmol/min/mg)
MCAD/*ACADM*	MCAD 1*^b^*	c.[985A>G];[985A>G]	0.01	0.68 ± 0.15
		p.[K329E];[K329E]		
MCAD/*ACADM*	MCAD 2*^b^*	c.[985A>G];[985A>G]	0.02	0.68 ± 0.15
		p.[K329E];[K329E]		
SCAD/*ACADS*	SCAD	c.[1138C>T];[1138C>T]	0.05	0.75 ± 0.20
		p.[R380W];[R380W]		
VLCAD/*ACADVL*	VLCAD	c.[798_801delAGTT];[798_801delAGTT]	0	3.73 ± 0.90

SBCAD	SBCAD	Unknown	0.01	0.22 ± 0.06
IVD/*IVD*	IVD	c.[367G>A];[367G>A]	0.01	2.48 ± 0.77
		p.[G123R];[G123R]		
SCEH/*ECHS1*	SCEH 1*^b^*	c.[473C>A];[414+3G>C]	<9	379 ± 147
		p.[A158D];[126-138del]		
SCEH/*ECHS1*	SCEH 2*^b^*	c.[473C>A];[414+3G>C]	<9	379 ± 147
		p.[A158D];[126-138del]		
3-MGH/*AUH*	3-MGH	c.[637G>A];[637G>A]	0.01	3.84 ± 0.69
		p.[A213T];[A213T]		
MTP/*HADHB*	MTP 1*^b^*	c.[1390-515_-499del];[1390-515_-499del]	LCHAD: 9	74 ± 20
		r.[1389_1390ins96]	LCTH: 2.3	84 ± 13
MTP/*HADHA*	MTP 2*^b^*	c.[2027G>T];[2027G>T]	LCHAD: 0	74 ± 20
		p.[R676L];[R676L]	LCTH: 0	84 ± 13
SCHAD/*HADHSC*	SCHAD	c.[547-3_549del];	8	104 ± 29
		[547-3_549del]		
		r.[547_636del]		
SBCHAD/*HSD17B10*	SBCHAD	c.[388C>T];[388C>T]	0.13	7.3 ± 1.3
		p.[R130C];[R130C]		
SCKAT/*ACAT1*	SCKAT	c.[1163+2T>C];[1163+2T>C]	Ratio +K/−K = 1	Ratio +K/−K = 2.54 ± 0.97
		r.[1163_1164insGCAG]		
MTP/LCKAT	LCKAT	c.[185G>A];[1292T>C]	LCHAD: 38	74 ± 20
*HADHB*		p.[R62H];[F431S]	LCTH: 0.8	84 ± 13

a c., refers to coding DNA reference sequence; r., refers to RNA reference sequence; p., refers to protein reference sequence.

b The numbers associated with the defective enzyme refer to the specific patients used in the figures.

### MCAD is the only acyl-CoA dehydrogenase responsible for oxidation of PB-CoA

**Fig. 1.**
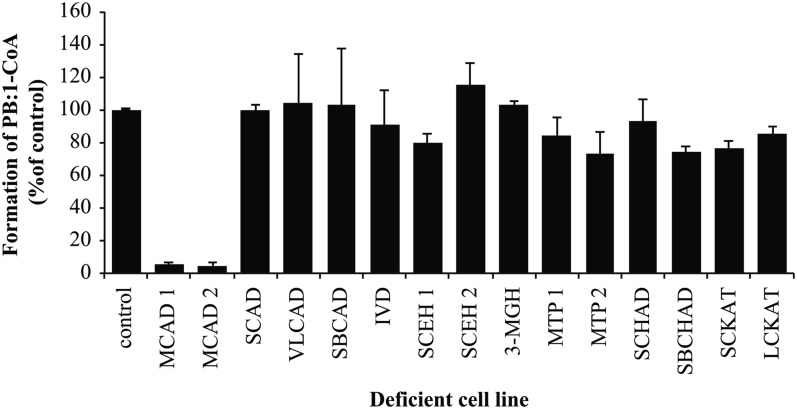
MCAD catalyzes the formation of PB:1-CoA. Control and deficient fibroblasts were incubated with PB-CoA for 30 min at 37°C. Formation of downstream β-oxidation products was analyzed using UHPLC. Product (PB:1-CoA) was quantified by measuring peak area on the chromatogram and normalizing to the activity found in control fibroblasts, which was set to 100%. Bars represent mean ± SD (n = 3), showing the amount of PB:1-CoA formed in different deficient cell extracts in comparison to control.

The first step in the β-oxidation is catalyzed by a member of the acyl-CoA dehydrogenase family. We tested five of the known acyl-CoA dehydrogenases that were most likely to react with short-chain fatty acid-derived substrate: short-chain acyl-CoA dehydrogenase (SCAD), MCAD, very-long-chain acyl-CoA dehydrogenase (VLCAD), short/branched-chain acyl-CoA dehydrogenase (SBCAD), and isovaleryl-CoA dehydrogenase (IVD). To identify which of these acyl-CoA dehydrogenases was responsible for the first step in the oxidation of PB-CoA to PA-CoA, we measured the formation of PB:1-CoA in fibroblast extracts that were deficient for these enzymes (Table 1), using PB-CoA as a substrate. Lysates from most deficient cell lines produced similar amounts of PB:1-CoA compared with controls, while cells lacking MCAD activity were fully deficient (**Fig. 1**). The complete lack of PB:1-CoA formation confirmed that MCAD was the only enzyme responsible for the first step in the PB-CoA β-oxidation cycle.

### PB:1-CoA hydratase activity is catalyzed by SCEH, LCEH, and 3-MGH

**Fig. 2.**
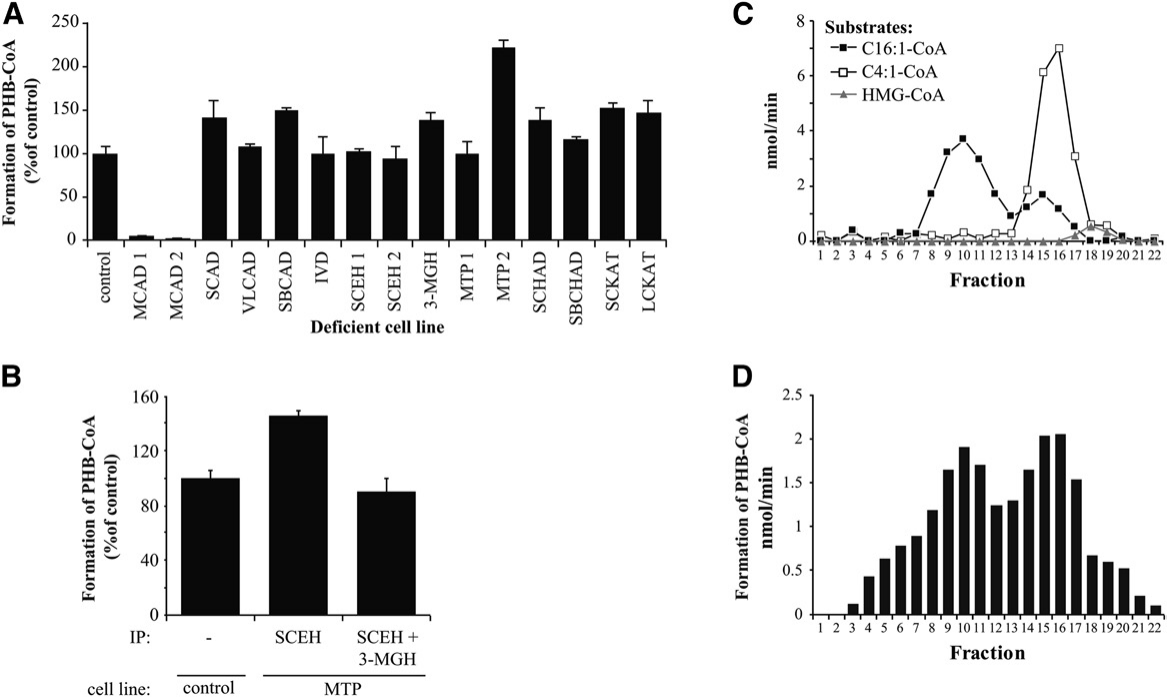
SCEH, LCEH, and 3-MGH catalyze the formation of PHB-CoA. A: PHB-CoA formation from PB-CoA was measured in control and β-oxidation-deficient fibroblasts. Control activity was set at 100%. B: PHB-CoA was measured in MTP-deficient (or LCEH-deficient) fibroblasts after immunoprecipitation to remove SCEH alone, or SCEH and 3-MGH together. The figure represents formation of PHB-CoA after incubation of multiple deficient lysates with PB-CoA in comparison to control. Bars represent mean ± SD (n = 3). C: Enoyl-CoA hydratases were separated by gel filtration. Twenty-two fractions were collected and analyzed for hydratase activity with C16:1-CoA (LCEH), C4:1-CoA (SCEH), or HMG-CoA (3-MGH) as substrate. D: Gel filtration fractions were incubated with PB:1-CoA to determine which hydratases are capable of catalyzing the hydration of PB:1-CoA to PHB-CoA. The amount of PHB-CoA formed is presented.

We employed a similar approach to determine the enzyme responsible for the second step in PB-CoA metabolism. To this end the conversion of PB:1-CoA to PHB-CoA was studied (**Fig. 2A**). This conversion is catalyzed by a member of the enoyl-CoA hydratase family: SCEH, LCEH, and 3-MGH. Formation of PHB-CoA from PB-CoA was normal in cells that were lacking either SCEH, LCEH, or 3-MGH activity, and was only blocked when using MCAD-deficient fibroblasts, as these could not synthesize the substrate for the hydratase reaction.

This suggested that PB:1-CoA was a substrate for not just one enzyme, but for two or more enzymes, and when one of these was deficient, affinity for the remaining hydratases was high enough to convert PB:1-CoA to PHB-CoA. To clarify this possibility, we removed specific enoyl-CoA hydratases from the lysate by immunoprecipitation and measured residual activity in the supernatant. When SCEH was removed from MTP-deficient lysates, PHB-CoA was still being formed (Fig. 2B). Because PB:1-CoA could be a suitable substrate for all three hydratases, we also removed the third hydratase (3-MGH) with immunoprecipitation. Despite the triple deficiency of SCEH, MTP, and 3-MGH, PHB-CoA was still being formed (Fig. 2B), suggesting the existence of a fourth enoyl-CoA hydratase whose activity could convert PB:1-CoA into PHB-CoA.

To determine which enoyl-CoA hydratase was capable of metabolizing PB:1-CoA to PHB-CoA, we performed gel filtration to separate mitochondrial hydratases by size and then determined the hydratase activity in all the different fractions. We collected 22 fractions and determined the presence or absence of SCEH, 3-MGH, and LCEH. LCEH is a part of MTP, a heterooctamer consisting of four α and four β subunits (400 kDa) and eluted in fractions 8–16, while SCEH eluted in fractions 14–17 and 3-MGH in fractions 18 and 19 (Fig. 2C). Incubation of these fractions with PB:1-CoA showed that all three mitochondrial hydratases were capable of transforming PB:1-CoA to PHB-CoA (Fig. 2D). In addition to these established hydratase activities, however, we also found PHB-CoA formation in fractions 4–7 (Fig. 2D). This supported the notion that at least one additional enzyme was present in cell homogenates that was capable of metabolizing PB:1-CoA.

### SCHAD and LCHAD catalyze the 3-hydroxyacyl-CoA dehydrogenase activity in PB-CoA oxidation

**Fig. 3.**
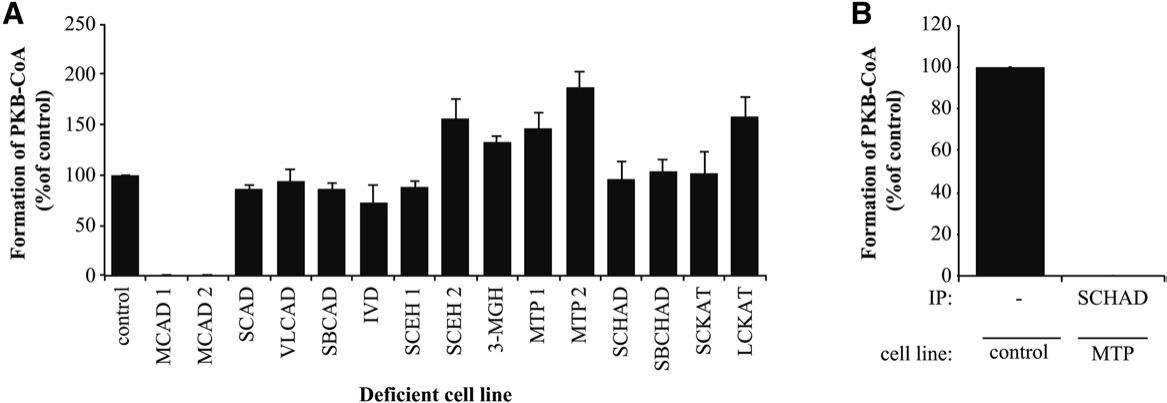
SCHAD and LCHAD catalyze the formation of PKB-CoA. A: Deficient cell lines were incubated with PB-CoA and metabolites were analyzed by UHPLC. The product, PKB-CoA, was quantified by measuring peak area on the chromatogram and normalizing to the activity found in control fibroblasts. Control activity was set at 100%. Bars represent mean ± SD (n = 3). B: From MTP-deficient cell extract, we removed SCHAD by immunoprecipitation and these lysates were incubated with PB-CoA. The figure shows formation of PKB-CoA compared with control.

To investigate the third step in the enzymatic conversion of PB-CoA, we focused on cell lines deficient in 3-hydroxyacyl-CoA dehydrogenase: SCHAD, LCHAD, and short branched-chain hydroxyacyl-CoA dehydrogenase (SBCHAD). Formation of PKB-CoA did not differ between control cells and cell lines deficient in any of the three 3-hydroxyacyl-CoA dehydrogenases (**Fig. 3A**), suggesting overlapping activities. We hence performed immunoprecipitation to remove multiple enzymes from the lysate. When we removed SCHAD from MTP-deficient cells, which lack LCHAD activity, we observed no formation of PKB-CoA (Fig. 3B), suggesting that both SCHAD and LCHAD could metabolize PHB-CoA to PKB-CoA.

### LCKAT is the 3-ketoacyl-CoA thiolase responsible for the final step in β-oxidation of PB-CoA

**Fig. 4.**
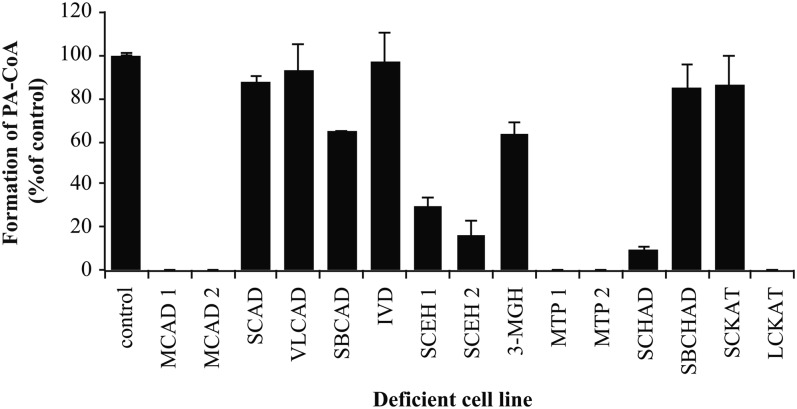
LCKAT catalyzes the formation of PA-CoA. To determine whether specific enzyme deficiency prevents formation of PA-CoA, incubations of deficient cell extracts with PB-CoA were performed and product formation (PA-CoA) was analyzed by UHPLC. Results were compared with control and are presented as percentage. Bars represent mean ± SD (n = 3).

The fourth and final step of the β-oxidation cycle is completed by an enzyme from the 3-keto-thiolase family, which in mitochondria consists of short-chain ketoacyl-CoA thiolase (SCKAT) and LCKAT as part of MTP. In the MTP-deficient cells, PHB-CoA and PKB-CoA were normally synthesized from PB-CoA, but there was no synthesis of PA-CoA (**Fig. 4**). This suggested that LCKAT was the only enzyme responsible for the last step in β-oxidation of PB-CoA. We confirmed this in cells with an isolated deficiency of LCKAT (23) (Fig. 4). Furthermore, cells from SCKAT-deficient patients showed normal PA-CoA synthesis, confirming that this enzyme was not involved in the final step of this pathway (Fig. 4).

## DISCUSSION

For several decades, PA has been used as a therapy for patients who suffer from hyperammonemia. The use of PA diminished because of its taste and smell and was replaced by the more acceptable PB (4), and more recently the use of a glycerol triester of PB to prevent toxicity of sodium load from using the sodium-salt form of PB (24). More recently, PB was proposed as a therapy for different human conditions, such as cancer and other metabolic diseases. Despite its increased use in medicine and the fact that PB was already used as a metabolic substrate in the initial experiments that led to the discovery of fatty acid oxidation by Franz Knoop in 1904 (19), the enzymology of PB metabolism has remained ill-understood.

**Fig. 5.**
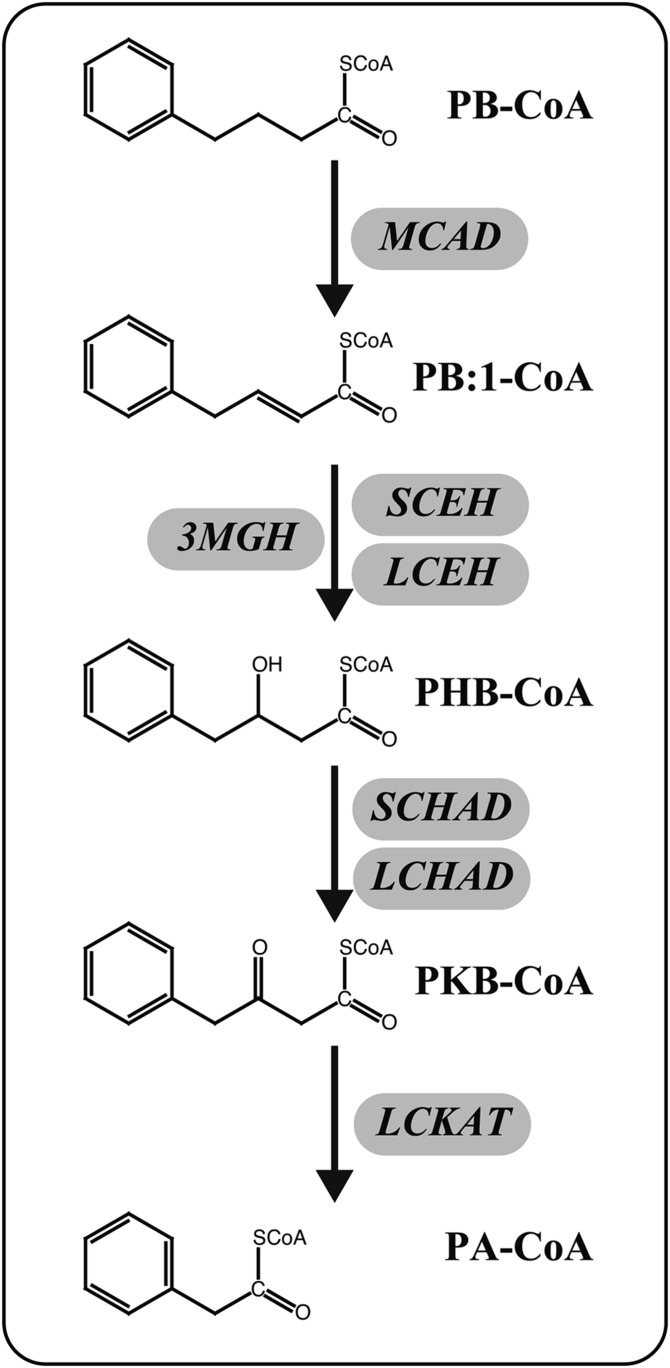
Schematic overview of PB-CoA oxidation. The enzymes that are capable of sequentially converting PB-CoA into PA-CoA are shown.

In order to elucidate the enzymology of the β-oxidation pathway of PB, we set out to determine the enzymes that were involved in this pathway. We used cell lines that were deficient in one of the β-oxidation enzymes, and measured accumulation of PB metabolites after incubation with PB. With this method, we first confirmed that MCAD, indeed, catalyzed the first step in the degradative pathway of PB (20) and demonstrated that LCKAT performed the last step, whereas the two intermediate steps could be mediated by several enzymes (**Fig. 5**). In fact, the second step, hydration, was not only catalyzed by SCEH, LCEH (as a part of MTP), and 3-MGH, but also by a fourth, yet uncharacterized, enzymatic activity. This could be due to the fact that these experiments were performed in cell homogenates. As a consequence, the homogenate contained not only mitochondrial, but also peroxisomal, β-oxidation enzymes. It is possible that PB:1-CoA is also a substrate for one or more of these enzymes. Based on gel filtration elution time, however, we could not determine the enzyme from the peroxisomes, as it appeared to be too large for any of the peroxisomal enzymes that are known so far (25). Finally, we could not exclude that other (yet unknown) enzymes might have a role in converting PB or its metabolites in vivo. First, we observed that a deficiency of, for instance, MCAD led to a strong, but incomplete, block in PB:1-CoA formation. While this most likely represented residual activity of the enzyme, we could not exclude that another dehydrogenase was active. A second consideration was the fact that certain enzymes involved in PB oxidation may not be expressed in fibroblasts.

The findings we present here could have implications for the therapeutic use of PB. Both PB and PA have been shown to conjugate to glutamine and serve to remove excess nitrogen in urea cycle-deficient patients (26), and both drugs induce differentiation in hematopoietic (27, 28) and solid (29, 30) tumor cell lines. In other studies, however, PB and PA induce different effects. For instance, PB is more potent at inhibiting proliferation and inducing apoptosis (30, 31), and some studies even suggest that PA has no significant impact on either proliferation or apoptosis of malignant cells (28, 32). Considering these differential effects, the activity of PB and PA could be determined by its metabolic fate. If for a specific clinical condition PB is the active compound, β-oxidation catalyzed by the enzymes described here leads to inactivation of the drug, but if PA is the relevant drug then β-oxidation will transform PB into the active compound PA. Regardless of the mode of action, conversion of PB can act as a modifying factor in its therapeutic use, increasing the importance of understanding the PB β-oxidation pathway.

In addition to the therapeutic efficacy that may be modified by PB β-oxidation, this pathway could also have implications for the drug’s toxicity. Even though PB has been tested successfully for cancer, motor neuron diseases, hemoglobinopathies, and cystic fibrosis (33), some patients have experienced dose-limiting toxicity (34). This is particularly relevant for conditions that require high doses of the drug, which could lead to neurocortical toxicity, possibly attributable to accumulation of the metabolite, PA (34, 35).

By identifying enzymes involved in the β-oxidation of PB, we have a better understanding of its metabolism and, with this knowledge, we should be able to increase the efficiency of the drug and reduce the occurrence of adverse effects. This is something that deserves further exploration in the near future.
